# Testicular Cancer Incidence among Obstructive Sleep Apnea (OSA) Patients: South Korean National Health Insurance Data

**DOI:** 10.3390/cancers15133273

**Published:** 2023-06-21

**Authors:** Marn Joon Park, Kyung-Do Han, Jae Hoon Cho, Ji Ho Choi

**Affiliations:** 1 Department of Otorhinolaryngology-Head and Neck Surgery, Inha University Hospital, School of Medicine, Inha University, 27 Inhang-ro, Jung-gu, Incheon 22332, Republic of Korea; parkmarnjoon@gmail.com; 2 Department of Statistics and Actuarial Science, Soongsil University, 369 Sangdo-ro, Dongjak-gu, Seoul 06978, Republic of Korea; hkd@ssu.ac.kr; 3 Department of Otorhinolaryngology-Head and Neck Surgery, School of Medicine, Konkuk University, 120-1 Neungdong-ro, Gwangjin-gu, Seoul 05030, Republic of Korea; 4 Department of Otorhinolaryngology-Head and Neck Surgery, College of Medicine, Bucheon Hospital, Soonchunhyang University, 170 Jomaru-ro, Bucheon 14584, Republic of Korea

**Keywords:** testicular cancer, sleep apnea, obstructive sleep apnea, incidence, national health programs

## Abstract

**Simple Summary:**

Obstructive sleep apnea (OSA) increases the risk of numerous cancers. Nevertheless, testicular cancer prevalence in OSA patients has not been documented. Based on KNHIS data, this study examines OSA’s effect on testicular cancer incidence. A total of 152,801 newly diagnosed male adult OSA patients and 764,005 controls were studied. Even after confounding correction, OSA’s HR for testicular cancer was 1.58 (95% CI: 0.92–2.60). The subgroup analysis showed a 3.39 (95% CI: 1.08–10.06) HR for testicular cancer in those over 65. The 20–40 and 40–65 age groups had no significant HR. OSA may not affect testicular cancer in adults in general. However, those over 65 may be more susceptible to OSA-related testicular cancer than younger individuals.

**Abstract:**

Obstructive sleep apnea (OSA) has been linked to an increased risk of acquiring many types of cancer. No data on the prevalence of testicular cancer in OSA patients have been reported in the literature. The goal of the present investigation is to find out the impact of OSA on the incidence of testicular cancer based on the Korea National Health Insurance Service (KNHIS) dataset. A cohort of adult male patients newly registered with OSA in the KNHIS data from 2007 to 2014 who had no history of any previous cancer diagnosis was included. The main outcome measure was newly diagnosed testicular cancer in the National Medical Expenses Support Program. The control group was set at five times larger than the OSA group, and it was matched with age and sex. The cumulative incidence and hazard ratio (HR) for the development of testicular cancer were compared between the OSA and control groups. Further subgroup analysis was conducted in the three different age groups. In the study period, a total of 152,801 male adult patients newly diagnosed with OSA were included, whereas 764,005 individuals were recruited as the control group. The HR of OSA for developing testicular cancer was 1.58 (95% confidence interval [CI]: 0.92–2.60), showing no significant HR regardless of confounding adjustment. However, the subgroup analysis revealed a significantly increased HR to develop testicular cancer of 3.39 (95% CI: 1.08–10.06) in groups aged more than 65, whereas the age ranges of 20–40 and 40–64 showed no significance (1.19 (0.44–2.75) and 1.27 (0.50–2.80), respectively). OSA may not influence the incidence of testicular cancer in the general adult population. However, compared to younger males, males over 65 may be more susceptible to OSA when it comes to developing testicular cancer.

## 1. Introduction

The prevalence of obstructive sleep apnea (OSA) is increasing globally over the past decades [1]. Among South Korean adults, there has been a reported prevalence of 21.6% of individuals having high risk of OSA in 2022 [2]. OSA patients exhibit intermittent hypoxemia (IH) with or without hypercapnia along with sympathetic activation [3]. OSA therefore acts as an independent risk factor for many cardiovascular [3] and even endocrine and metabolic disorders [4]. Intriguingly, it has been suggested that OSA may be linked to a number of cancerous tumors in humans [5]. In many previous studies, it was shown that patients with severe nocturnal intermittent hypoxemia who were untreated independently had a significantly increased risk of developing all types of malignancies [6,7,8]. Furthermore, although many previous researchers have revealed that OSA may act as an independent risk factor for developing breast [9], thyroid [6], prostate [10], and colon cancer [11], no previous studies in the literature have aimed to analyze the impact of OSA on testicular cancer development.

Testicular cancer is a relatively uncommon malignant neoplasm, accounting for only 1% of male malignancies [12]. However, it is the most common solid malignant tumors in males aged between 15 and 35, and it has been shown to have a better prognosis than other types of cancer [13]. More than 95% of testicular cancer patients have germ cell tumors [12], and their incidence has slowly increased over the past 50 years [14,15]. 

In South Korea, where the current study took place, the crude and age-standardized rate (ASR) of incidence for testicular cancers were reported to be both 0.5% in 2015 and 1.3% in 2018, respectively [16,17]. Among all of the urologic malignancies, testicular cancers exhibit the best prognosis, showing a 5-year survival rate of 94.2% in the Korean population, which is the highest survival rate compared with other urologic cancers such as prostate (92.3%), renal (79.9%), and bladder (75.4%) [16]. 

Previously, it has been well established that patients with cryptorchidism, family history of testicular cancer, infertility have an increased risk of developing testicular cancer [18,19]. Although other acquired and environmental factors, such as in utero viral exposures and testicular trauma [20], have been hypothesized to be associated with testicular tumors, none of them have been found to account for the rising incidence of testicular malignancies. 

Interestingly, there has been some reports regarding the low testosterone levels [21,22] and altered pituitary–gonadal functions in OSA patients [23]. Moreover, an experimental study has revealed an association between chronic intermittent hypoxia, which is similar to the changes following OSA and leads to infertility [24], and decreased testosterone synthesis [25]. However, neither the effect of OSA on the development of testicular cancer nor the relationship between OSA and testicular cancer have been reported in any of the prior literature. By analyzing the Korea National Health Insurance Service (KNHIS) database, the authors aimed to determine the effect of OSA on the incidence of testicular cancer in a large cohort over a 10-year period.

## 2. Materials and Methods

### 2.1. Ethical Declaration

This study was approved by the Institutional Review Board (IRB) of Inha University Hospital (Investigation No.: 2022-11-041). The IRB has reviewed and approved the study design and exempted the study subjects from providing informed consent. The authors have strictly adhered to research standards while being formally supervised by the IRB.

### 2.2. Study Population; the Korea National Health Insurance Service (KNHIS) Database

This research was conducted using KNHIS information. Since the year 2000, the South Korean government has required all citizens to be registered and covered by the KNHIS for medical services [26]. After submitting a formal dissertation protocol and receiving ethical approval from the official review committee, the KNHIS provides access to the archived data. Each individual enrolled in the KNHIS is assigned a unique resident registration number, eliminating the prospect of duplication or omission during data analysis. The KNHIS examines both inpatient and outpatient claims, as well as demographic information, clinical diagnoses, medical expenses, and diagnostic or therapeutic interventions. The KNHIS classifies and stratifies all medical claims according to the Korean Standard Classification of Diseases, sixth edition (KCD-6), which is a modified variant of the International Classification of Diseases, 10th Revision (ICD-10).

By searching the KNHIS dataset for the operational identifier for OSA (G47.30), patients diagnosed with OSA were identified. Each patient’s age and income level were compiled. In addition to the asserted insurance data, the patient’s medical history, including diagnoses of hypertension, diabetes, and dyslipidemia was collected. Table 1 provides additional information on the operational definition and search requirements for each disease.

### 2.3. Study Population; Inclusion and Exclusion Criteria 

**Figure 1:**
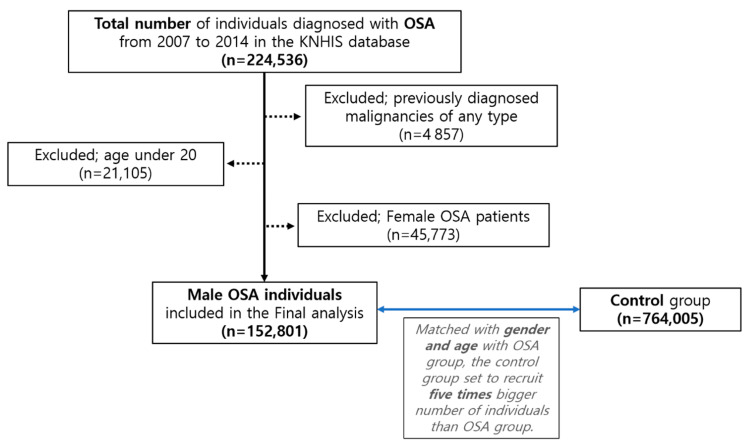
Study design and enrollment of male OSA and control groups. Using data from the KNHIS, a flow diagram details how participants were selected for the obstructive sleep apnea group and the control group for the final analysis. Abbreviations: KNHIS, Korea National Health Insurance Service; OSA, obstructive sleep apnea.

This study included male individuals older than 19 who received a new OSA diagnosis (G47.30) between 2007 and 2014. This observational study’s primary endpoint was the incidence of newly diagnosed testicular cancer in newly diagnosed OSA patients. Patients newly diagnosed with OSA during that time frame were enrolled in a cohort, and the cohort’s claimed insurance data were retrospectively reviewed until the end of 2015, resulting in an observation period of over seven consecutive years. The presence of newly diagnosed testicular cancer (ICD-10 code C 33 or C 34) was retrospectively reviewed in the National Medical Expenses Support Program registry during the cohort period (Table 1). The patient’s testicular cancer diagnosis date and whether or not they were removed from the KNHIS upon demise were censored. The time interval between the diagnosis of OSA and testicular cancer, defined as a ‘person-year at risk’ for developing a newly onset testicular cancer, was calculated for all subjects included in the study. A control group was recruited to compare the cumulative risk of testicular cancer incidence in OSA patients. Patients without an OSA diagnosis were selected as controls using a propensity score that matched their gender and age. We have employed the “exact matching method” for matching the control group’s gender and age. To elucidate, individuals without an OSA claim enrolled in the KNHIS who share the same sex and age as individuals with an OSA claim were selected at random in five times greater numbers. Thus, it was determined that the total number of participants in the control group would be five times that of OSA patients. Prior to enrollment, patients with any type of malignant tumor (searching for any operation codes of malignant neoplasm) were precluded from both the OSA and the control groups. Figure 1 depicts a flowchart detailing the specifics of the OSA cohort and the control group selection procedure. 

### 2.4. Statistical Analysis

A descriptive statistical analysis was conducted on a variety of demographical and clinical data. Depending on the categories of each clinical variable, the OSA and control groups were compared using a Student’s *t*-test or a chi-square test. A cumulative incidence diagram was created to compare the incidence rate of newly diagnosed testicular cancer between the OSA group and the control group. In both the OSA and control groups, the hazard ratio (HR) for testicular cancer development was calculated using three distinct Cox proportional hazards models. In model A, the covariate was not taken into account when calculating the HR. In model B, only age was used to modulate the HR. In model C, age, income, hypertension, diabetes, and dyslipidemia were taken into account as confounding factors when calculating the adjusted HR. Using logistic regression analysis, subgroup analyses were undertaken to determine the hazard ratio (HR) of OSA for developing testicular cancer in three age groups: 20 to 40, 40 to 65, and over 65. To estimate the significance of differences in the HR between three age subgroups, a p for interaction was calculated using the likelihood-ratio testing method. All statistical analyses were two-tailed, and the results were presented with a confidence interval (CI) of 95%. All statistical analyses were conducted using SAS version 9.4 (SAS Institute, Cary, NC, USA) or R version 3.2.3 (The R Foundation for Statistical Computing, Vienna, Austria). 

## 3. Results

### 3.1. Clinical and Demographic Characteristics between the OSA and Control Group

During the study period of January 2007 through December 2014, a total of 49,570,064 individuals were registered in the KNHIS in 2007, as depicted in Figure 1. In the study period, 152,801 male patients were newly diagnosed with OSA. A total of 764,005 males were recruited for the control group. The mean standard deviation (SD) interval for follow-up in the cohort was 4.5 ± 2.3 years.

Table 2 presents various demographic and clinical diagnoses for the male OSA and control groups. As the ages of OSA patients and controls were matched during the recruitment of the control group, there were no statistically significant age differences between the two groups (*p*-value = 1.0). In contrast, the distribution of other demographic variables varied significantly between the two categories. All other comorbidities such as diabetes, hypertension, and dyslipidemia were shown to be more prevalent among OSA patients. The income level of OSA patients was substantially greater than that of the control group.

### 3.2. The Effect of OSA on the Development of Testicular Cancer

**Figure 2:**
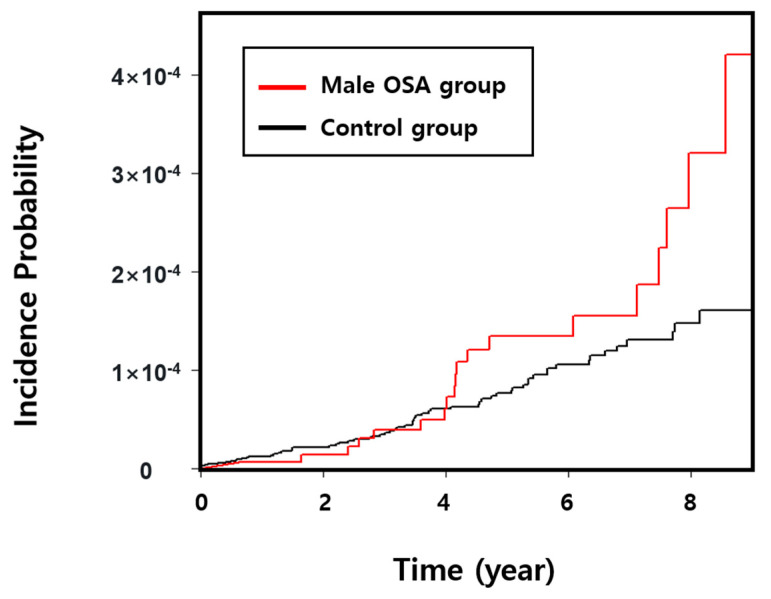
Comparison of the cumulative incidence of testicular cancer between the male OSA group and the control group. In a seven-year cohort, there was no statistically significant difference in the cumulative incidence of testicular cancer between the OSA and control groups. Abbreviations: OSA, obstructive sleep apnea.

There were no statistically significant differences between the male OSA group and the control group in terms of the incidence of testicular cancer. The incidence of newly diagnosed testicular cancer following OSA diagnosis did not differ statistically between OSA and control groups, as shown in Figure 2’s cumulative incidence plot for newly onset testicular cancer.

The HR calculated from the Cox proportional hazard model to evaluate the impact of OSA in developing testicular cancer did not show statistical significance, regardless of adjustments with confounding (Table 3). Model A, which is a model without any adjustments, revealed an HR of 1.58 (95% CI; 0.92–2.60). Models B and C, which were adjusted for age (model B), and various comorbid diseases along with age (model C), showed an adjusted HR of 1.58 (95% CI; 0.92–2.60) and 1.55 (95% CI; 0.89–2.56), respectively.

However, when it came to the subgroup analysis, according to three different age groups, the age more than 65 group showed a significantly increased adjusted HR of 3.39 (95% CI; 1.08–10.06) (Table 4). By contrast, the age between 40 to 65 group (adjusted HR of 1.27 [95% CI; 0.50–2.80]) and age between 20 to 40 group (adjusted HR of 1.19 [95% CI; 0.44–2.75]) showed no such significance in the adjusted HR.

## 4. Discussion

Our findings reveal that adult male OSA patients did not have a higher cumulative incidence or risk of newly diagnosed testicular cancer compared to an age-matched control group. These results persisted after confounding factor adjustments. However, in the analysis of subgroups according to each gender, patients with OSA who were above the age of 65 and male exhibited a statistically significantly elevated adjusted HR of 3.39 compared with the control group. On the other hand, patients who were relatively young and were either between the ages of 40 and 65 or between the ages of 20 and 40 did not indicate such significance.

A growing number of researchers are taking an interest in sleep-related breathing disorders, which include obstructive sleep apnea (OSA), as an independent risk factor for the development of different malignant cancers in the human body [5,8,27,28]. The presence of chronic intermittent hypoxemia in OSA may hasten the development and progression of tumors in a variety of organs by triggering a variety of responses in the neuroendocrine, cardiovascular, and respiratory systems [29,30,31,32]. As presented in many previous studies, including meta-analyses, a relative risk between 1.26 and 1.53 has been reported for OSA as an independent risk for developing all cancers studied in various nations and ethnicities [8,27,33]. In addition, the findings of previous studies suggested a dose–response connection between obstructive sleep apnea and the occurrence of cancer [33,34]. In a study published in 2015, the total cancer incidence and death rates were up to three times greater in those with severe OSA compared to those with mild to moderate OSA [34]. However, these findings were not always consistent in every other study, as the findings of several other studies contradicted this conclusion [5,7]. In the previous study on cancers of different organs, obstructive sleep apnea (OSA) was found to have a substantial impact on the development of prostate, breast, colorectal, renal cell carcinomas, and pancreatic cancers, as OSA acted as a significant risk factor for developing such cancers [7,35]. Overall, the findings of prior clinical and epidemiological investigations point to the potential and clinical influence that OSA may have on the prevalence of a number of different malignancies.

However, the epidemiology and clinical features of testicular cancers are very different from those of other cancers. Most testicular cancers arise in males in their late 30 s to 40 s [36,37,38]. Currently, according to the World Health Organization classification in 2016, testicular cancers are subdivided into three types: type I testicular germ cell tumor (TGCT), type II TGCT, and type III TGCT, depending on the histological features and biomarker specificities [39]. Age at diagnosis is highly relevant for these subtypes [40]. Type I TGCT, known as prepubertal type teratoma, includes yolk sac tumors and arises from pre-erased embryogenic germ cell lines [41]. Type II TGCT originates from fully erased embryogenic germ cells, which include seminomas and non-seminomas [42]. On the other hand, type III TGCT develops from a fully erased paternal germ cell lineage [39]. While type I TGCTs mostly arise before puberty, most of the type II TGCTs occur following puberty, and type III TGCTs are known to always occur after puberty; most cases are predominantly diagnosed in the elderly population [40,43]. Although we were not able to identify the subtypes of the testicular cancers in our study subjects, there is a possibility that the testicular cancer patients included in our study might mostly consist of type II and III TGCT tumors, owing to the fact that our study included only adult patients. Moreover, the significantly high risk of developing testicular cancer in males aged over 65 might suggest that OSA might only increase the risk of type III TGCTs, but it might not significantly increase the risk of developing type I or II TGCTs. Because the KHNIS data do not include the histological subtypes of the testicular cancers, we were not able to obtain the testicular cancer subtypes. To clarify the hypothesis raised according to our results, it would be interesting to investigate which subtypes are significantly associated with OSA in the future.

Increased cancer rates in the OSA population have been theorized to be caused by two key characteristics: sleep fragmentation (SF) and IH [44]. Frequent hypoxia followed by normoxia in IH simulates a scenario comparable to the reperfusion damage in tissues that endured ischemia stress [45]; however, the precise mechanism of IH on carcinogenesis is yet unknown. Endothelial cells in vascular structures exposed to chronic IH may produce reactive oxygen species, which may predispose normal tissues to carcinogenesis [8]. Another potential mechanism by which IH promotes carcinogenesis is through upregulating many hypoxia-inducible factors (HIFs) in multiple organs [5,46]. Furthermore, several studies reveal that SF may lead to a heightened sympathetic nervous system response, persistent inflammation, and altered immune cell activities, all of which may promote carcinogenesis in a number of body systems [28,47]. In addition, it should be acknowledged that testicular cancers exhibit a very unique epidemiology, molecular changes, and carcinogenesis, suggesting that a further study should be conducted in order to support the possible causality of OSA and testicular cancer, particularly in the elderly population [38,40]. As Cheng et al. described different molecular and genetic alterations according to the subtypes of testicular cancers [40], it might serve as an area of interest to investigate whether the IH during OSA might alter the molecular changes that are closely related to those changes known to be associated with type III TGCTs in the future. Moreover, many researchers have reported low testosterone levels and hypogonadism among testicular cancer patients, especially in those who are obese and having metabolic syndromes [21,22]. As both OSA and hypogonadism are more prevalent in obese individuals [48], it might be an interesting area to investigate how OSA affects the human endocrine systems in terms of gonadal hormones among other hormones as well.

Our research’s strengths include its substantial statistical power, its adjustments for possible confounding factors such as age and a variety of concomitant conditions, and its subgroup analysis based on the ages of the patients who were enrolled in the study. Although testicular cancer is very well known to be a rare malignant tumor [14], based on the fact that all citizens of South Korea are required to have health insurance via the National Health Insurance Service, we were able to obtain well-assessed data on a large scale [26]. In addition, when compared with studies that used a cross-sectional or case-control design, the fact that both OSA and control cohort groups were monitored for seven consecutive years may substantially support the causal relationship between the carcinogenic potential of OSA and testicular cancer in our data. In addition, the availability of a subgroup analysis on the risk of testicular cancer associated with OSA according to three distinct age groups brings out the most important aspects of our research. To the best of the authors’ knowledge, there was no prior research that discussed or addressed the effect that OSA has on testicular cancer; our study is the first to assess the influence that OSA has on the development of testicular cancer. As a consequence of the fact that our research is based on large power and a long observation period in a cohort, our data may cautiously support the possibility that a causal association may be formed in the elderly male OSA patients with an elevated risk for the development of testicular cancer.

Although our study presented some unprecedented findings with a seven-year cohort on a nation-wide scale, the authors acknowledge some limitations that must be declared. First, we were not able to identify the subtypes of testicular cancers or the histological, molecular, and genetic mutations in each patient diagnosed with testicular cancer in our study. In addition, we were not able to consider the known associated factors with testicular cancer, i.e., familial history of testicular cancers, contralateral testicular cancers, and cryptorchism in each patient, which are some known major risk factors for testicular cancer [19]. Second, owing to the fact that testicular cancer is a very rare tumor, even with a long observation period in a national-wide study, there were some weakness in the power of the obtained sample size, as the range of 95% CI of adjusted-HR in the age more than 65 showed a wide range from 1.08 to 10.06. Third, we did not assess the collinearity between the observed variables such as age, hypertension, diabetes, etc. In order to more precisely evaluate the testicular cancer risk in OSA individuals, while adjusting these clinical variables, the degree of possible interactions between those variables shall be assessed and corrected. Fourth, the KNHIS dataset solely furnished information on patients who received a diagnosis of OSA without any accompanying particulars concerning the severity of OSA (as indicated by the apnea–hypopnea index, etc.), the degree of obesity as represented by the body mass index, or whether the patients underwent any form of treatment for OSA. Consequently, the analysis of the dose–response relationship between the severity of obstructive sleep apnea (OSA) and the confounding influence of obesity on the development of testicular cancer in individuals with OSA was not feasible. In future research, it would be advisable to take into account both the degree of obstructive sleep apnea (OSA) and the individual’s body mass index (BMI) in order to provide further clarity on this topic. Lastly, to accurately estimate the impact of OSA on the incidence of testicular cancer, it is necessary to account for an adequate quantity of time. However, our dataset and study design were not sufficient to elucidate to calculate the requisite amount of time for carcinogenesis of testicular cancer according the to the OSA. Despite the limitations, our results provide a remarkable addition to the knowledge base in terms of OSA and cancer development, especially in reporting the previously unmentioned findings.

## 5. Conclusions

Generally, OSA does not raise the risk of testicular cancer incidence in the Korean adult population. However, when it comes to subgroup analysis according to age, OSA may impact testicular cancer development in male OSA patients aged more than 65 but not in those aged less than 65.

## Figures and Tables

**Table 1:** Methods and criteria for searching for patients with each ailment in the KNHIS database.

Name of Each Disease	Search Protocols of Each Disease
Inclusion criteria	
Obstructive sleep apnea	Patients who at least once had an ICD-10-CM code of G47.3 declared for them in the KNHIS dataset
Primary endpoint	
Testicular cancer	Patients who at least once had an ICD-10-CM code of C62 declared for them in the KNHIS dataset
Confounding factor analyses for various diseases	
Diabetes	Individuals in the KNHIS dataset who received at least one prescription for an anti-diabetes medicine in the study period and who had an ICD-10 diagnosis code of E11–14
Hypertension	Individuals in the KNHIS dataset who received at least one prescription for an anti-hypertensive medicine in the study period and who had an ICD-10 diagnosis code of I10, I11, I13, or I15
Dyslipidemia	Individuals in the KNHIS dataset who received at least one prescription for an anti-dyslipidemic medications in the study period and who had an ICD-10 diagnosis code of E78

ICD, International Classification of Diseases; KNHIS, Korea National Health Insurance Service.

**Table 2:** Clinical and demographic characteristics of the adult male OSA patients and control group.

N (%) or Mean ± SD	Male OSA Patients *N* = 152,801	Control Group *N* = 764,005	*p*-Value *
Mean follow-up duration (years)	4.5 ± 2.3	4.5 ± 2.3	1.0
Mean age (years)	45.6 ± 13.2	45.6 ± 13.2	1.0
Aged between 20 to 40	53,786 (35.2)	268,930 (35.2)	1.0
Aged between 40 to 65	81,595 (53.4)	407,979 (53.4)	
Aged more than 65	17,420 (11.4)	87,096 (11.4)	
Income (the lowest quintile)	26,434 (17.3)	170,373 (22.3)	<0.001
Diabetes	11,307 (7.4)	45,840 (6.0)	<0.001
Hypertension	34,686 (22.7)	110,781 (14.5)	<0.001
Dyslipidemia	28,115 (18.4)	65,704 (8.6)	<0.001

OSA, obstructive sleep apnea; SD, standard deviation. * *p*-value calculated with *t*-test or chi-square test, depending on the variable characteristics.

**Table 3:** The hazard ratio (HR) of OSA for developing testicular cancer.

	Number	Event	Crude Rate (Event/Number) (%)	HR Calculated in Model A * HR (95% CI)	HR Calculated in Model B ^+^ Adjusted HR (95% CI)	HR Calculated in Model C ^‡^ Adjusted HR (95% CI)
Control group	764,005	60	0.0079	1 (reference)	1 (reference)	1 (reference)
OSA group	152,801	19	0.0124	1.58 (0.92–2.60)	1.58 (0.92–2.60)	1.55 (0.88–2.57)

Abbreviations; CI, confidence interval; HR, hazard ratio; OSA, obstructive sleep apnea. * Model A was derived using Cox proportional hazards analysis with no confounding variables. **^+^** Model B was derived using Cox proportional hazards analysis with only age. **^‡^** Model C was derived using Cox proportional hazards analysis adjusted with the subjects’ age, income level, diabetes, hypertension, and dyslipidemia.

**Table 4:** The hazard ratio (HR) of OSA developing testicular cancer in each age group.

	Age between 20 to 40		Age between 40 and 65		Age over 65	
	Number/ Event/ Crude Rate (%)	HR (95% CI) ^†^	Number/ Event/ Crude Rate (%)	HR (95% CI) ^†^	Number/ Event/Crude Rate (%)	HR (95% CI) ^†^
Control group	174,957/23/0.0131	1 (reference)	519,523/31/0.0060	1 (reference)	69,525/6/0.0086	1 (reference)
OSA group	44,622/7/0.0157	1.19 (0.44–2.75)	89,621/7/0.0078	1.27 (0.50–2.80)	18,558/5/0.0269	3.39 (1.08–10.06)
*p* for interaction *	0.016					

Abbreviations; CI, confidence interval; HR, hazards ratio; OSA, obstructive sleep apnea. * *p*-value for interaction with age in the subgroup analysis was calculated using the likelihood-ratio testing method. ^†^ HR calculated with logistic regression analysis adjusted with the subjects’ age, income level, diabetes, hypertension, and dyslipidemia.

## Data Availability

All data relevant to the study are included in the article.
